# Advances and Challenges in Spinal Cord Injury Treatments

**DOI:** 10.3390/jcm13144101

**Published:** 2024-07-13

**Authors:** Mohammed Ali Alvi, Karlo M. Pedro, Ayesha I. Quddusi, Michael G. Fehlings

**Affiliations:** 1 Institute of Medical Science, University of Toronto, Toronto, ON M5S 1A8, Canada; mohammed.alvi@uhn.ca (M.A.A.); karlo.pedro@uhn.ca (K.M.P.); ayesha.quddusi@mail.utoronto.ca (A.I.Q.); 2 Department of Surgery and Spine Program, University of Toronto, Toronto, ON M5T 1P5, Canada; 3 Division of Neurosurgery, Krembil Neuroscience Centre, Toronto Western Hospital, University Health Network, Toronto, ON M5T 2S8, Canada

**Keywords:** spinal cord injury, timing of surgery, neuroregeneration, surgery, neuroprotection, clinical practice guidelines, biomarkers

## Abstract

Spinal cord injury (SCI) is a debilitating condition that is associated with long-term physical and functional disability. Our understanding of the pathogenesis of SCI has evolved significantly over the past three decades. In parallel, significant advances have been made in optimizing the management of patients with SCI. Early surgical decompression, adequate bony decompression and expansile duraplasty are surgical strategies that may improve neurological and functional outcomes in patients with SCI. Furthermore, advances in the non-surgical management of SCI have been made, including optimization of hemodynamic management in the critical care setting. Several promising therapies have also been investigated in pre-clinical studies, with some being translated into clinical trials. Given the recent interest in advancing precision medicine, several investigations have been performed to delineate the role of imaging, cerebral spinal fluid (CSF) and serum biomarkers in predicting outcomes and curating individualized treatment plans for SCI patients. Finally, technological advancements in biomechanics and bioengineering have also found a role in SCI management in the form of neuromodulation and brain–computer interfaces.

## 1. Introduction

Our understanding of the intricacies and pathways underlying the pathophysiology of SCI has evolved significantly over the past three decades. This has led to several paradigm shifts concerning the management of SCI. Specifically, the importance of mitigating secondary injury mechanisms following the initial physical trauma has been highlighted by several landmark studies [1,2,3]. Several candidate strategies have been studied that target various components of this chemical and molecular injury cascade [1,2].

Despite these advances, the neurological prognosis for most SCI patients remains poor. Regaining lost neurologic function, particularly quadriparesis or quadriplegia in cases of cervical SCI and paraparesis or paraplegia in cases of thoracolumbar SCI, continues to be a significant challenge. The factors that impede regeneration after SCI include axonal degeneration, inhibitory factors in the post-injury micro-environment, particularly the chronic astroglial scar, and loss of neuronal and oligodendroglial populations [4].

From a public health perspective, neurotrauma involving the brain and spine has been shown to be the leading cause of disability around the world [5]. Optimizing treatment outcomes can be aided by customizing management to identify persons living with SCI who have the highest potential to gain from neuroprotective and/or neuroregenerative approaches [6].

In the current review, the authors first examine the difficulties associated with conducting research on SCI, including the challenges when assessing the severity of SCI as well as the limitations of traditional statistics when evaluating treatment efficacy. The authors then explore advances in surgical management and promising recent translational advances for SCI.

## 2. Challenges in SCI Research

SCI represents a complex and heterogenous clinical entity characterized by diverse presentations and outcomes. The interplay of factors such as the injury level, severity, and associated comorbidities contributes to the wide variability observed among SCI patients, impacting their motor, sensory, psychological and social outcomes.

The evolving landscape of SCI’s epidemiology reflects its inherent heterogeneity. While significant progress in public health efforts has reduced the incidence of SCIs from motor vehicular collisions (MVCs), sports and occupational trauma in high-income countries, a notable increase in SCI cases among the elderly, particularly due to falls, has been observed. A study by Aarabi et al. demonstrated that across a 17-year span, the average age and proportion of SCI cases resulting from falls increased significantly, while those arising from MVCs and sports-related injuries declined [7]. This demographic shift is further corroborated by a US-based study reporting the rising mean age of SCI patients from 40 years in 1993 to 50 years in 2012 [8]. Projections suggest that the majority of traumatic SCI patients will exceed 70 years of age by the year 2032 [9].

The complexity of SCI is likewise evident in the multitude of scales employed to assess and grade SCI patients across various domains. Such complexity demands innovative solutions to represent the clinical profiles and outcomes of these patients. Relying solely on a traditional singular scale such as the American Spinal Injury Association (ASIA) Impairment Scale (AIS) may be restrictive, given the breadth of factors influencing SCI outcomes.

Embracing adaptive clinical trial designs and multiparametric outcome representation enables researchers to capture the nuanced nature of SCI presentation and treatment responses [10]. Trajectory modeling offers a dynamic framework for analyzing patient outcomes over time, revealing unique clinical profiles and prognostic indicators. This approach has revealed targeted clinical relationships between variables and patient outcomes among those with incomplete and complete SCI. Additionally, machine-learning algorithms, including supervised- and unsupervised-learning techniques, provide powerful tools for extracting meaningful insights from complex SCI datasets [11]. By leveraging large-scale data repositories and advanced computational methods, machine leaning can facilitate personalized risk stratification and treatment allocation in SCI management. These innovative methodologies both enhance the scientific rigor of research as well as hold promise for translating research findings into meaningful improvements in SCI care and outcomes.

## 3. Recent Surgical Advances and Guidelines

### 3.1. Timing of Surgery

Strong evidence that surgical decompression within 24 h of the initial traumatic insult significantly improves patient outcomes at the 6-month follow-up was shown by the seminal multicenter, multinational, prospective cohort study known as the “Surgical Timing in Acute Spinal Cord Injury Study (STASCIS)” [12]. When compared to patients who underwent surgical decompression after 24 h, patients who underwent earlier decompression were more likely to experience an improvement of at least two grades on the AIS scale. Additionally, this research showed that surgical decompression can be carried out safely before the 24 h mark, without increasing the risk of complications or mortality.

Subsequent clinical trials, conducted both in North America and internationally (including the United Kingdom, Iran, China and Pakistan), have shown the superiority of early surgical intervention vs. late surgical decompression [13,14]. In essence, there is a higher likelihood of better outcomes in patients who receive surgical decompression within 24 h as compared to those undergoing late surgery. A more recent study assessed the effectiveness of surgical decompression prior to 24 h. The results of this study also showed that surgical decompression within 24 h of the initial damage was linked to better sensorimotor recovery at the 1-year follow-up. Early surgery was significantly correlated with improvements in both light touch and pinprick scores. Improvement in the mean motor scores was also linked to early surgery in the same study. There was a persistent decrease in motor recovery when surgery was performed between 24 and 36 h after injury [15,16].

Although there is strong evidence that surgery performed within 24 h enhances patient outcomes, there is still debate over the exact cutoff period. The role of early and ultra-early time periods has been investigated in recent studies. At the 6-month follow-up, the ultra-early surgery (before 8 h) group showed superior results as compared to the 8–24 h cutoff cohort in a single-center study [17]. In contrast, another study by Bock et al. found that the 4 h and 4–24 h cutoffs for surgical decompression did not differ significantly in terms of the neurological improvement [18].

The SCI-POEM (Prospective, Observational, European Multicenter Cohort Study) is a recently published study on the timing of surgery in SCI. The primary endpoint of this multicenter European study was improvement in the lower extremity motor function score (LEMS) at 12 months. The observed difference with the late surgery group was not statistically significant (*p* = 0.065 and 0.245, respectively), despite the fact that a trend toward better improvement in the LEMS was linked with early surgery (<12 h) in both the unadjusted and adjusted analyses [19]. When interpreting the study findings, it is important to note that an imbalance in covariates persisted between the early and late surgical groups, despite employing propensity score matching and multiple imputations of missing data. This imbalance may have contributed to the non-significant findings of this study. To overcome these limitations, a true randomized controlled trial (RCT) would be required. However, this endeavor poses challenges related to ethics, logistics, and the fundamental issue of achieving clinical equipoise.

#### Guidelines for the Timing of Surgical Decompression

The 2017 AO Spine SCI guidelines provide evidence-based recommendations for the timing of surgical intervention in patients with SCI, utilizing 24 h as a threshold to distinguish between early and late decompression [20]. Based on a systematic evaluation of the evidence, a weak recommendation was made to provide early surgery as an option for adult SCI patients, regardless of the injury severity [20].

Since the publication of these guidelines, numerous studies evaluating the efficacy of early versus late surgical decompression on various outcomes after SCI have emerged. A 2021 meta-analysis that combined data from four sizable datasets involving more than 1500 patients found that patients who were decompressed within 24 h had higher ASIA motor score improvement and AIS conversion [15]. In 2022, in light of the emergence of several new studies regarding the timing of surgery, the Guidelines Development Group (GDG) reconvened to synthesize updated clinical practice guidelines (CPGs). In this updated CPG based on a synthesis of the literature [21], a strong recommendation for surgical decompression within 24 h of SCI was made (Table 1) [22]. However, further research is essential, particularly concerning the definition and potential effectiveness of ultra-early surgery. The “24-h cut-off” utilized in the STASCIS trial was based on the approximate median duration from injury to surgical decompression, which was deemed logistically and medically feasible [12]. Nonetheless, the secondary injury cascade follows a temporal pattern, suggesting a biological rationale for the potential benefits of decompression even sooner than 24 h after injury. Despite this, the GDG could not definitively specify, given the current state of knowledge, what constitutes ultra-early decompression or the impact of earlier time thresholds (i.e., <4, <8 h) on neurological recovery.

It is important to note that the varying definitions of what constitutes ultra-early surgery in the literature create a challenge for definitively answering the timing of surgery question [12,24,25,26,27]. Moreover, the timing of surgery after an injury differs from the timing of surgery after hospital admission. To address these issues, it is recommended that future research adopt uniform definitions of ultra-early surgery to facilitate the pooling of data. Moreover, the time from injury to surgical decompression can be treated as a continuous variable in post hoc studies employing sophisticated analytical techniques, which may yield valuable insights [28]. In fact, a dose–response link between the timing of surgical intervention and neurological recovery has been proposed using this approach [15].

### 3.2. Hemodynamic Management

In addition to surgical decompression, controlling the mean arterial blood pressure (MAP) offers clinicians an opportunity to optimize recovery and prevent further ischemic damage to the spinal cord after injury. Patients are closely monitored for respiratory insufficiency, cardiac dysfunction, and systemic hypotension post-injury [29]. Hypovolemia from concurrent bleeding and neurogenic shock increases the risk of systemic hypotension in SCI patients, which further aggravates secondary injury to the spinal cord. Reduced MAP has been suggested to have a major impact on the spinal cord through alterations in the oxygen supply to susceptible tissue, a decrease in spinal cord perfusion, and a worsening of secondary damage. The damaged spinal cord’s compromised vascular responsiveness and loss of autoregulation renders it especially vulnerable to systemic hypotension. According to Ryken et al., volume expansion and deliberate blood pressure increases were believed to enhance the neurological outcomes as well as lower mortality and morbidity in patients with SCI [29].

In order to synthesize the effects of goal-directed interventions aimed at optimizing spinal cord perfusion on neurological recovery and adverse events, as well as the effects of monitoring techniques, perfusion ranges, pharmacological therapies, and treatment duration, Evaniew et al. (2020) conducted a high-quality systematic review of the current evidence base [29]. Pertinent interventional and observational research, both prospective and retrospective, was included in this study. The analysis highlighted several limitations of previous systematic reviews, specifically that none evaluated the possibility of bias in the included studies or employed the GRADE (Grading of Recommendations, Assessment, Development and Evaluations) methodology to appraise the quality of the available evidence. Moreover, no other evaluations have examined the effects on neurological outcomes of combined spinal cord perfusion pressure (SCPP) and MAP.

This systematic review also noted that across studies, it remained unclear how increasing MAP would affect neurological recovery [30]. Several included studies showed that (i) the motor scores did not differ between patients who received vasopressors and those who did not, and (ii) the MAP decreases were not linked to changes in the ASIA motor scores [31,32]. While MAP support was found to be effective in other included investigations, these studies had lower sample sizes and MINORS (Methodological Index for Non-Randomized Studies) scores, which suggests a higher risk of bias [30]. Additionally, there are little data to support the hypothesis that vasopressor support of MAP is linked to a higher incidence of arrhythmias, cardiac damage, and posterior reversible encephalopathy syndrome [30]. The approach to hemodynamic care for acute SCI must take all of these aspects into account.

While the use of SCPP has been shown to produce better neurological outcomes as measured by the AIS [30], using intradural catheters to evaluate SCPP is an invasive monitoring method that can increase the risk of meningitis or CSF leak [33]. Reviewing the strength of the evidence for the possible benefits of elevating MAP or SCPP, weighing the potential risks associated with each strategy, and assessing the viability and cost-effectiveness of these treatments are all crucial when establishing recommendations.

#### Guidelines for Hemodynamics for SCI

In addition to the timing of decompression, the AO Spine GDG also updated the guidelines for hemodynamic management after SCI. The GDG recognized the very poor quality of the data that were available and developed weak recommendations for maintaining the mean artery pressure (MAP) for three to seven days post-SCI, within a range of 75–95 mmHg on the higher end [23]. Given the current state of the research, no recommendations on the use of a particular vasopressor or inotrope were provided. This is a significant deviation from the 2013 AANS/CNS guidelines [29], which suggested that the MAP be kept between 85 and 90 mmHg for seven days (Table 1). However, the GDG concurred that there was insufficient evidence to support a seven-day course of treatment for all patients, and the literature did not consistently support a neurological benefit with this range. Therefore, these guidelines reflect the uncertainty that exists in the field regarding the neurological consequences of specific MAP targets. They also emphasized the importance of high-quality data to better delineate the connection between MAP targets and neurological outcomes. The University of California San Francisco (UCSF) group has been collecting high-frequency (q1 min) physiologic and hemodynamic data (such as MAP values through an arterial line) with an automated computerized system for many years; as more institutions adopt electronic medical records, they will likely be able to access similar large datasets and apply sophisticated analytical techniques to better understand the relationship between particular MAP targets and neurological outcome [34].

The GDG concurred that there were insufficient data to formulate guidelines on SCPP. Additionally, due to the limited centers actively researching this parameter, providing a recommendation was deemed unfeasible. Understanding the differences between measuring the intrathecal/CSF pressure distal to the SCI in the lumbar cistern using a pressure/drainage catheter system that is familiar to most clinicians and using a pressure catheter inserted locally during surgery (as described by Papadopoulos et al.) [35] will potentially address the knowledge gaps pertaining to the use of SCPP for future research [31,36,37,38]. The work of Papadopoulos et al. has already demonstrated that the “intraspinal” pressure at the site of damage may significantly exceed the “intrathecal” pressure in the lumbar cistern when the injured spinal cord expands against the dura [39]. The method for achieving “complete decompression” (as previously described) is clearly related to this aspect of hemodynamic management, as is the growing understanding of the role of spinal cord swelling and occlusion of the subarachnoid space when the injured spinal cord abuts against the dura, potentially causing an increase in intraspinal pressure.

Lastly, while research on the ideal MAP or SCPP is still in progress, there is a crucial need for methods to track physiological reactions at the actual site of injury. According to research conducted by Kwon et al. [40], monitoring the physiology and metabolism of the injury site intradurally in response to hemodynamic control is feasible. This monitoring approach can provide valuable insights into the real processes that occur within the spinal cord following an injury. Efforts are underway to develop comparable strategies using epidural technology, such as near-infrared spectroscopy, which utilizes near-infrared light to non-invasively examine spinal cord tissue [41,42].

### 3.3. Adequacy of Decompression and Expansile Duraplasty

Although there are no means to reverse the primary mechanical damage to the cord, research has shown promise in minimizing the molecular cascades causing the secondary injury [43,44,45,46]. A potentially salvageable zone known as the penumbra surrounds the site of initial damage, where secondary injury starts. Localized bleeding at the site of injury is one of the earliest signs of rupture of the microvasculature in the central gray matter. As the bleeding progresses, edema develops and the spinal cord swells [39,47,48,49,50,51]. Within one to two hours following trauma, injured tissue can be detected on T2-weighted and short tau inversion recovery magnetic resonance imaging (MRI) [52,53,54,55]. In individuals with cervical SCI, the rate of intramedullary lesion (IML) expansion varies based on the severity of the damage. It can be as slow as 200 micrometer/hour in AIS C patients and as high as 900 micrometer/hour in AIS A and B patients [56,57].

The term “adequacy” of decompression has been defined in terms of the intramedullary lesion length (IMLL) expansion. Aarabi et al. conducted a study in which they found that the degree of spinal cord decompression, assessed by the amount of perimedullary cerebrospinal fluid (CSF) seen on the postoperative MRI, seemed to have an impact on the AIS grade conversion for patients having surgery for SCI. The conversion rate was 18.5% in individuals without sufficient decompression, whereas the chance of conversion with complete spinal cord decompression over all the IMLL segments was 58.9%. Patients with inadequate decompression had an IMLL of 100.3 mm, while those with adequate decompression (with laminectomy) had an IMLL of around 62.4 mm. For patients who had adequate decompression throughout all the observable segments on the postoperative MRI, the odds of AIS conversion were found to be significantly higher. The authors also found that quantitatively, the probabilities of AIS grade conversion decreased by 40% for every 10 mm increase in the IMLL [57,58]. The same team then looked at which surgical approaches may provide adequate decompression of the spinal cord. The authors found that in individuals with motor complete cervical SCI, laminectomy was linked to an increased likelihood of complete spinal cord decompression. Notably, the success rate of decompression improved with increased levels of laminectomy performed [57].

These results suggest that among patients with cervical SCI, the spread of secondary spinal cord swelling may extend caudally below T1 and rostrally toward the brainstem, which may drastically increase the risk of morbidity and mortality. Since decompression may have a significant impact on the AIS grade conversion, planning a surgical operation to optimize the likelihood of complete spinal cord decompression may be just as important as the timing. The concept of the combined effects of surgical decompression degree and timing of the cervical spinal cord following trauma continues to be important, as evidenced by recent investigations [12,20,59,60,61,62].

### 3.4. Expansile Duraplasty

The first recommendation for the use of expansile duraplasty was made by Werndle et al. [63], in connection with monitoring spinal cord perfusion after decompression to check for SCI. Following injury, there is increased edema and swelling of the spinal cord parenchyma, causing it to compress against the surrounding dura. This causes an abrupt and localized rise in intraspinal pressure (ISP), ultimately inhibiting autoregulation. Impaired autoregulation is associated with increased SCPP (e.g., hyperperfusion) and decreased SCPP (e.g., hypoperfusion), which may result in further SCI akin to intracranial dynamics. Consequently, it has been suggested in some reports to utilize a posterior midline dural incision made longitudinally for duraplasty purposes after bone decompression. While this practice may enhance the ISP as well as SCPP and provide additional decompression to the spinal cord, its benefits must be weighed against other potential risks, including infection and CSF leak [64]. A prospective, phase III, multicenter randomized controlled trial is currently underway to investigate if, among acutely injured cervical SCI patients, the addition of dural decompression in combination with bony decompression will improve muscle strength at 6 months using the AIS motor score as the primary outcome. The DISCUS (Duraplasty for Injured Cervical Spinal Cord with Uncontrolled Swelling) study (NCT04936620) is currently recruiting patients and is estimated to be completed in 2026 [65].

### 3.5. Therapeutic Hypothermia

Therapeutic hypothermia has been recommended as a supplement to the conventional care of SCI due to its neuroprotective properties. It is thought to reduce bleeding, edema, and pressure while interrupting glutamate excitotoxicity, oxidative stress, and inflammation. Pre-clinical and early clinical trials investigating the use of therapeutic hypothermia for SCI suggest it may have a role as a neuroprotective strategy. Following an injury, therapeutic hypothermia can be used locally or systemically through endovascular, extradural, or intradural methods. Based on data from human trials, Shin et al. conducted a recent meta-analysis and found that, while systemic hypothermia was not statistically significant, it was preferable to local hypothermia in terms of the improved neurological outcomes.

Further research is required on the means of inducing therapeutic hypothermia and determining the optimal temperature target. While physical techniques like cooling fluid have been most commonly examined for SCI and other conditions, including stroke, pharmacological techniques to produce therapeutic hypothermia are a novel area of investigation. In a rodent model of SCI, dihydrocapsaicin and physical cooling produced similar results in terms of the neurological and histological outcomes, as well as reaching the target temperature of 33.0 ± 1.0 °C.

Although hypothermia has demonstrated potential as a neuroprotectant, several important questions remain to be answered before it is incorporated into clinical practice. These include determining the optimal mode of administration, the ideal rate of cooling, and the optimal initiation time after injury. Multicenter randomized trials are needed to provide definitive answers to these questions. 

## 4. Translational Advances

### 4.1. Pharmacological Advances

A key element of the pathophysiology of SCI involves neurodegeneration, which is characterized by progressive loss of neurons and synapses. Numerous chemical and molecular events contribute to degeneration, including oxidative stress, apoptosis, inflammation, and excitotoxicity [66]. Since neurons have limited capacity to regenerate, neuronal damage is frequently irreparable and irreversible [67]. Significant work has been conducted to study candidate pharmacological agents with the potential to enhance repair and regeneration after SCI. The possibility of using currently available medications with neuroprotective or pro-regenerative capabilities makes “drug repurposing” a very attractive prospect in the context of SCI. Since these medications have already been evaluated and approved for other medical disorders, repurposing them can save significant capital and time. The utilization of drug repurposing holds promise in expeditiously identifying pharmaceuticals suitable for clinical trials, thereby facilitating the identification of agents that effectively enhance outcomes for individuals with SCI [68]. In the following section, we will discuss some of the biological and pharmacological agents currently being evaluated in preclinical and clinical trials for SCI (Table 2).

#### 4.1.1. Riluzole

Riluzole, a benzothiazole anticonvulsant, has been shown to have neuroprotective properties, likely due to its mechanism of blocking sodium channels, which in turn helps to reduce excitotoxic injury [76,77,78]. In a rodent cervical spine hemisection model, Satkunendrarah et al. showed that riluzole treatment increased the preservation of glutamatergic synapses and motor neurons caudal to the lesion site, resulting in improved locomotor and respiratory function [79]. Riluzole has been evaluated in numerous clinical trials as a potential therapy for SCI. Variable levels of peak plasma concentration were identified in a phase I trial (NCT00876889) evaluating the safety and pharmacokinetics of riluzole in acute traumatic SCI. No major side effects were observed; however, some patients did experience mild-to-moderate elevations in liver enzymes. Patients in the riluzole group were reported to demonstrate greater improvements in the average motor score [70,73,74,75,77,80,81,82].

The Riluzole in Spinal Cord Injury Study (RISCIS), a subsequent phase IIB/III clinical trial, was discontinued because of enrollment issues during the COVID-19 pandemic. The trial’s goal was to assess improvements in the motor scores of the International Standards for Neurological Classification of Spinal Cord Injury (ISNCSCI). Due to insufficient power secondary to early termination, the authors were unable to achieve the pre-set endpoint to evaluate efficacy. However, all cervical SCI patient subgroups (AIS grades A, B, and C) treated with riluzole demonstrated improvements in functional recovery, which were part of the preplanned secondary analyses.

#### 4.1.2. Anti Nogo-A Antibody

Fish and amphibians have regenerable CNS axons, whereas regeneration in mammalian CNS axons is inhibited. The main reason behind this lack of capability to regenerate is the presence of myelin proteins in mammals that impede CNS axon regeneration. A number of these proteins have been found to cause growth cone collapse. One of these proteins is Nogo-A, which binds to the Nogo receptor, activating the Rho-ROCK pathway [76,83]. Antibodies against Nogo may inhibit the Rho-ROCK pathway and facilitate axonal regeneration following SCI. In a macaque model, Freund et al. delivered the anti-Nogo antibody using an implanted osmotic pump and showed improvements in axonal sprouting and dexterity after cervical-level injuries [76,84,85]. Acute SCI patients who received the anti-Nogo-A antibody (ATI355) through intrathecal injection demonstrated improvements in their motor scores and had satisfactory antibody tolerance [72,76]. Anti-Nogo antibody therapy is presently being tested in both acute and chronic SCI through the RESET trial (NCT03989440) and Nogo Inhibition in Spinal Cord Injury (NISCI) clinical trial (NCT03935321), respectively [76].

#### 4.1.3. Anti-RGMa Antibody

Repulsive Guidance Molecule A (RGMa) binds to the Neogenin receptor, triggering the RhoA-Rho kinase pathway, which leads to the inhibition of axon development. Following SCI, RGMa is elevated in myelinated areas as well as at the lesion site and is expressed by both neurons and oligodendrocytes. RGMa-targeting antibodies have been developed and tested in pre-clinical models of SCI, demonstrating enhanced neuronal survival, axon regeneration, dexterity, and locomotion in rodent and primate models of SCI. In the ELASCI trial (NCT04295538), a human anti-RGMa monoclonal antibody called Elezanumab (ABT-555) is being studied for safety, effectiveness, and improvements in upper-extremity motor function for acute SCI [86].

#### 4.1.4. Methylprednisolone Sodium Succinate (MPSS)

Methylprednisolone sodium succinate (MPSS) functions as a corticosteroid and antioxidant to reduce inflammation. MPPS improves spinal cord blood flow by blocking lipid peroxidation and lowering calcium influx [87]. In the NASCIS II trial, patients’ motor scores increased by 4.8 points (*p* = 0.03) compared to the placebo group when MPSS was administered within 8 h of injury. While the rates of morbidity and mortality in each treatment group were comparable, there was a notable increase in wound infections with MPSS (7.1%) compared to placebo (3.6%) [88]. In NASCIS III, the group that received 48 h of MPSS showed an additional 6 points of motor improvement compared to the 24 h MPSS category for patients whose therapy was started 3 to 8 h after injury. It was suggested that patients receiving MPSS within the first 3 h of SCI continue treatment for 24 h, while patients who received MPSS 3 to 8 h after injury continue treatment for 48 h [89]. According to a 2017 clinical practice guideline based on a review of the available evidence for MPSS in SCI, it was suggested that a 24 h MPSS infusion be considered for patients with SCI within 8 h of injury [90].

### 4.2. Precision Medicine

For many years, efforts have been made in SCI research to improve the capacity to reliably predict neurological and functional outcomes in SCI patients. Imaging, serum, and CSF biomarkers have been examined as potential modalities to predict the neurological outcomes and the severity of neural injury.

#### 4.2.1. Imaging Biomarkers

Accurate and unbiased neuroimaging measurements may have the potential to predict pathophysiological alterations in the spinal cord and explain neurological heterogeneity after injury. Determining imaging biomarkers that correlate with neurological function and recovery may aid in prediction and patient stratification.

Martin et al. recently described a radiographic white matter injury biomarker that corresponds with focal motor and sensory abnormalities. Specifically, it measures the signal intensity of white to gray matter on T2*-weighted imaging [91]. In addition, the neurological deficits of patients at baseline have been shown to have some associations with the IMLL, maximum spinal cord compression (MSCC), and maximum canal compromise (MCC) [85,86]. Patients with sensorimotor complete SCI have been shown to have more extensive acute lesion lengths, MSCCs, and MCCs, with the latter being correlated with baseline AIS grades. Similarly, baseline motor scores are associated with more prominent MSCC and MCC [92,93].

When paired with an automatic segmentation technique, conventional MRI can be used to quantify the lesion severity that results from acute SCI. Based on the “deepseg” technique in the SpinalCord Toolbox, the axial T2w-derived lesion volumes have been found to correlate well with the LEMS upon hospital release [94,95,96]. Most importantly, when it comes to segmentation of the spinal cord and lesion, the automatic segmentation tool based on 2D convolutional neural networks utilized in this study compares favorably with other methods that are currently available. Thus, automatically identifying and segmenting the intramedullary cyst from traditional T2w images may enhance the accuracy of MRI-based outcome prediction as well as the objectivity of lesion measurement [96].

Imaging biomarkers may also be paired with plasma cytokines to predict outcomes. Telegin et al. recently described a 10-grade scoring scale to assess the severity and extent of the damage to the cord in an experimental SCI model based on the initial MRI. The score was composed of four components, including the area of hyperintensity, area of hypointesity, the ratio between the two and the presence of syringomyelia cyst. This score, together with levels of cytokines such as IL-1α, IL-1β, IFNγ and TNFα, was found to have strong correlation with the functional outcomes in the authors’ injury model [97].

As a cutting-edge MRI method, diffusion tensor imaging (DTI) is sensitive to water movement and provides quantitative information about the integrity of the axons that run both parallel and transverse to their course [98,99]. Following acute SCI, aberrant diffusion indices (i.e., enhanced radial diffusivity (RD), mean diffusivity (MD), decreased axial diffusivity (AD) and fractional anisotropy (FA)) have been linked to axonal degeneration and demyelination [100]. These indices can be used to monitor intramedullary microstructural changes. Additionally, they have been linked to neurological injury and recovery trajectories. More specifically, a decrease in AD indicates damaged axons, while an increase in RD denotes demyelination, and a decrease in FA is observed in both scenarios [101]. Acute cervical SCI patients exhibit reduced FA but not MD levels for the total white matter (WM), sensory tracts, and motor tracts at the injury level when compared to healthy controls and one level caudal to the injury [102]. Decreases in the apparent diffusion coefficient following SCI parallel increases in the FA values, reflecting less degenerated axons from the acute stage to 6 months post-SCI, which are furthermore associated with AIS grades over the 6-month follow-up period. The DTI parameters measured in rostral lesion proximity show better correlation with clinical measures at different timepoints than those measured at the lesion epicenter. These parameters can also be used to discriminate between injury severity and neurological recovery. At the lesion level, conventional MRI characteristics, such as the lesion length and hemorrhagic contusion, outperform higher values of MD and AD, which indicate less axonal integrity and are predictive of lower motor scores and walking ability at 6–12 months post-SCI [103,104].

#### 4.2.2. CSF Biomarkers

The levels of specific molecules have been found to be elevated in the CSF following traumatic brain injury (TBI) and SCI [105,106]. Thus, molecular analysis of CSF from SCI patients has the potential to uncover biomarkers of clinical relevance that may help diagnose, assess severity, and predict outcomes. The clinical feasibility of this approach, however, remains challenging as CSF is not routinely collected after SCI [107]. Furthermore, obtaining CSF through a lumbar puncture is invasive and may have associated risks [108,109].

In a recent study consisting of 50 SCI patients, called the CAMPER (Canadian Multicentre CSF Monitoring and Biomarker Study) study, there was a significant difference in the CSF levels of GFAP, tau, IL-6, and S100b, at 24 h post-injury between ASIA A, B and C patients (NCT01279811) [110]. Furthermore, the CSF levels of these molecules were found to be associated with ASIA motor score improvement, particularly for patients with cervical SCI. In comparison to MRI-based biomarkers, CSF biomarkers may be more likely to identify distinct damage predictors and have a superior ability to predict neurological recovery [111].

Several other inflammatory mediators have been studied in CSF samples from patients with SCI. Studies have reported the CSF of SCI patients to contain higher levels of pro-inflammatory cytokines (e.g., IL-1β, IL-8, IL-16, TNF), chemokines (e.g., C-X-C motif chemokine (CXCL)-10, neutrophil attractant protein (NAP)-2, monocyte chemoattractant protein (MCP)-1), and growth factors (e.g., nerve growth factor (NGF)) [103,104,112,113] The clinical utility of these agents requires further investigation.

#### 4.2.3. Serological and Genetic Biomarkers

While CSF biomarkers may provide valuable information for SCI prediction or patient stratification, its clinical utility is hindered by the invasive nature of sample collection. As such, there is great appeal in studying serological or blood-based biomarkers. In clinical studies, higher concentrations of pro-inflammatory cytokines (TNF, colony stimulating factor-1, IL-2, IL-2R, IL-3, IL-6, IL 9, IL-16, IL-18) were present in the serum of SCI patients compared to those in the control group [105,106,107,108,109,110,114,115,116,117,118,119] Frost et al. (2005) found that 37 individuals with chronic SCI did not have higher levels of TNF or IL-6, in contrast to several earlier investigations [120]. SCI patients have also been shown to have elevated levels of the anti-inflammatory cytokine IL-10 and the IL-1 receptor antagonist (IL-1RA), in addition to a pro-inflammatory cytokine profile. In contrast, the levels of several pro-inflammatory cytokines (IL-1β, sIL-2Rα, IL-4, IL-5, IL-7, IL-13, IL-17, interferon-γ) and chemokines (macrophage inflammatory protein-1α and MIP-1β and granulocyte-macrophage colony stimulation factor) were significantly lower in acute SCI patients compared to non-SCI patients. In particular, the circulating levels of IL-10 were significantly higher in SCI patients than in non-SCI patients [121].

### 4.3. Regenerative and Cell-Based Therapies

Over the past few decades, cellular transplantation as a regenerative therapy for SCI has attracted a lot of attention. This is due to the fact that cell-based strategies may target several elements of the secondary injury cascade of SCI. For example, cell transplantation can provide trophic support, regulate the inflammatory response, repair damaged neuronal circuits, and regenerate denuded axons. Neural stem cells (NSCs), mesenchymal stem cells (MSCs), oligodendrocyte progenitor cells (OPCs), olfactory ensheathing cells (OECs), and Schwann cells are the most commonly examined cell types (Table 3) [1,122].

#### 4.3.1. Neural Stem Cells (NSCs)

Due to their tripotent self-renewing capacity, neural stem cells have been shown to improve functional recovery by myelinating denuded axons and encouraging tissue sparing in animal models of SCI [141]. NSCs significantly improve neurological function in preclinical SCI models, according to a 2016 meta-analysis (pooled SMD = 1.45; 95% confidence interval [CI]: 1.23–1.67; *p* < 0.001) [142]. Patients with thoracic AIS grades A–C and cervical AIS grades B or C SCI underwent intramedullary injections of human fetal CNS human stem cells (HuCNS-SC) as part of a phase II trial conducted by StemCells Inc. While positive trends toward the Upper Extremity Motor Score (UEMS) and Graded Redefined Assessment of Strength, Sensibility, and Prehension (GRASSP) motor gains were shown in the interim analysis of patients who were transplanted with HuCNS-SCs, the magnitude of the improvement was below the clinical efficacy threshold that the sponsor had set in order to support further development. Therefore, the trial was terminated early [123].

Stem cell transplantation may work synergistically with other therapies. For instance, transplantation of NSCs in combination with the delivery of chondroitinase ABC (ChABC), used to degrade the glial scar, was shown to improve recovery in animal models of SCI [143] (Table 3).

#### 4.3.2. Mesenchymal Stem Cells (MSCs)

Over the past few years, there has been increasing interest in bone marrow-derived stem cells as a possible therapeutic agent for a variety of illnesses. These cells have lower immunogenicity when allografted, and they release cytokines as well as exosomes that reduce inflammation [144]. In SCI, mesenchymal stem cells are thought to work by first migrating into the injured cord and then changing phenotypically to become neural cell phenotypes that allow factors promoting repair to be expressed rather than replacing damaged cells [145].

Multipotent MSCs can be extracted from readily accessible tissue, including fat, bone marrow, and skeletal muscle [146]. They also proliferate quickly, show little immunoreactivity upon allogenic transplantation, and remain alive after being cryopreserved in liquid nitrogen or at −80 °C [147,148,149]. This has prompted their translation into a number of areas, including arthritis [150], multiple sclerosis [151], and sepsis [152]. Through neurotrophic signaling and immunomodulation, MSCs have been demonstrated to greatly improve tissue sparing and stimulate angiogenesis in SCI [153,154]. Additionally, studies have suggested that MSCs injected directly into the spinal cord can control macrophage activity and encourage tissue sparing [155,156].

There are several active clinical trials evaluating the safety, efficacy, and dosage of MSCs produced from adipocytes. A phase I study of 1 × 10^8^ autologous adipose-derived MSCs administered intrathecally to patients with AIS grade A/B/C 2 weeks to 1 year after injury is being carried out at the Mayo Clinic (N = 10; clinicaltrials.gov identifier NCT03308565). The investigation is scheduled to be finished in June 2024 [127]. The effectiveness of injecting bone marrow-derived stem cells above, inside, and into the injury cavity, as well as the subdural space, was studied in a phase 3 experiment. At least 12 months after the injury, 16 patients with traumatic, cervical, sensory incomplete spinal cord injury (AIS grade B, with one patient having AIS grade A) were enrolled. There have been no reports of any negative transplant-related occurrences [157].

An additional source of MSCs is the umbilical cord (UC-MSCs). UC-MSCs are typically extracted from cord tissue, cord blood, or Wharton’s jelly, a gelatinous material found in the cord. Since umbilical cord tissue is easily obtained and often discarded, MSCs derived from it have been shown to be less susceptible to rejection, as indicated by a decreased risk of graft-versus-host disease [158]. The quantity of MSCs found in cord blood or placental tissues is lower than in adult sources, but they can be easily grown, and the tissue can be frozen and used for isolation at a later time [159]. It has also been demonstrated that MSCs obtained from the umbilical cord possess immunomodulatory qualities [160]. In a study by Moinuddin et al., the authors performed a T9 contusion and then randomized 12 female Sprague Dawley rats to either a control or rat UC-MSC group. By 14 weeks after their injuries, animals receiving rUC-MSC treatment demonstrated early and persistent motor recovery [161]. In order to evaluate allogeneic UC-derived MSCs for subacute and chronic SCI, a number of recently registered phase I/II trials (NCT03505034 [124]; NCT02481440 [125]; NCT03521323) [126] are currently accepting participants (Table 3).

#### 4.3.3. Oligodendrocyte Progenitor Cells (OPCs)

Oligodendrocyte progenitor cells (OPCs) are another cell source that has been studied in the treatment of SCI, given their potential to develop into myelinating oligodendrocytes. Based on pre-clinical studies, OPCs have been shown to improve motor recovery by reducing the cavitation volume, enhancing the white matter sparing, and increasing oligodendrocyte survival [162]. In the SCiStar trial, AIS grade A and B patients with subaxial cervical injuries received intramedullary injections of OPCs. The results showed that 95% of patients (21/22) recovered at least one motor level on one side, and 32% (7/22) recovered two or more motor levels on one side [128] (Table 3).

#### 4.3.4. Schwann Cells

Schwann cells of the peripheral nervous system (PNS) are myelinating cells that express growth-promoting proteins and act as a structural framework to direct developing axons and myelinate regenerating axons [163]. Immunosuppression is not required for the manufacture of autologous cell transplants, thanks to dependable cell culture techniques.

Schwann cells travel with axons throughout the process of neurodevelopment. These cells, together with the basal lamina components they release, form tubular bands during nerve healing. These tubular bands allow axons to either regenerate or contract, depending on whether they are surrounded by the extracellular matrix or the cytoplasm of non-myelinating Schwann cells. The ligands known to be involved in this process of nerve regeneration include the binding of laminin’s RGD peptide to axonal L1-NCAM. Gene expression alterations accompany the myelinating to non-myelinating phenotypic transition in Schwann cells. Schwann cells have the ability to revert to a stable myelinating subtype during nerve regeneration. The sparse integration of Schwann cells with astrocytes and oligodendroglia is one of the drawbacks of transplanting the cells into the central nervous system.

A phase 1 clinical trial was conducted recently in the US, which examined the safety of intramedullary transplanted autologous Schwann cells for complete thoracic SCI. The trial included six patients. Each patient’s sural nerve was used to extract cells, which were then preprocessed in vitro to ensure healthy cell proliferation. The cells were transplanted 30–60 days following damage; five million cells were transplanted in two patients, ten million cells in two more patients, and fifteen million cells in the final two patients when the dose was increased to evaluate the dose-dependent safety. Under ultrasound guidance, cells were transferred into the exposed damage epicenter using a stereotactic syringe-positioning device. In terms of safety, there have been no adverse effects associated with nerve harvesting or the transplant surgery. For AIS grade A to B patients, the clinical improvements reported in the study were within the typical range for those with thoracic SCIs. Subclinical improvements in the motor cortical connections were observed in neurophysiological investigations (motor evoked potentials and electromyography), with enhanced activity, particularly below the initial spinal level at which damage was discovered.

Through numerous international research efforts, the viability and safety of administering stem cells to the injured spinal cord are described in a systematic review of clinical trials. Nevertheless, the efficacy is still under verification. Research is currently ongoing to determine the best cell type and transplantation technique for lesion bridging and remodeling, lowering immunological rejection, as well as creating stable circuits [164] (Table 3).

### 4.4. Endogenous Stem Cells

In addition to the transplantation of exogenous cells, there is great appeal in harnessing the spinal cord’s own capacity for regeneration. The central canal region of the spinal cord contains a population of ependymal derived neural stem/progenitor cells that may possess stem cell-like properties. While normally quiescent, these cells become activated after SCI and primarily differentiate into astrocytes, with a smaller subset acquiring an oligodendrocytic fate. Several studies have demonstrated that these cells are important in promoting axon regeneration, providing beneficial trophic support and contributing to baseline functional recovery [130,131,132]. Current efforts aim to study strategies to further enhance the activation of endogenous ependymal derived neural stem/progenitor cells and direct their fate specification [133,134,135] (Table 3).

### 4.5. Biomaterials/Scaffolds

For targeted and sustained medication delivery, a variety of biomaterial scaffolds have been examined [150]. These have been shown to promote the development, survival, and plasticity of cells by introducing exogenous stem cells into the injured area as well as providing an environment and scaffold for the regeneration of endogenous circuits [165,166]. When injected into the spinal cord cavity, QL6, a biodegradable peptide, forms nanofiber scaffolds that decrease inflammation, apoptosis, and astrocyte lysis, leading to improvements in behavior as well as electrophysiology [167,168].

In the INSPIRE trial, 19 patients had open surgery to implant the Neuro-Spinal Scaffold 96 h after their injuries. At 24 months, there were no long-term neurological complications, and at 12 months and beyond, favorable AIS conversions were observed [136] (Table 3).

### 4.6. Upregulating Intrinsic Regenerative Potential

Reactivating intrinsic growth programs and promoting axon regeneration through the manipulation of neuronal intrinsic factors have been the focus of extensive research recently. Some of the primary intrinsic processes that govern axon regeneration are cytoskeletal dynamics, ion channels, and signaling pathways. We discuss a few advances in these areas here [169].

Following a mild spinal cord contusion in rats, it has been demonstrated that the microtubule-stabilizing medications Taxol and epothilone B promote microtubule polymerization and reduce fibrotic scarring, thereby inducing axon regeneration and improving function [170].

Neuronal survival and axon growth are regulated by intracellular signaling pathways, including DLK, JAK/STAT/SOCS3, and mTOR. A growing body of research indicates that adjusting these pathways may improve neuroplasticity and intrinsic growth competence following spinal cord injury [171,172,173,174,175].

Voltage-gated calcium channels’ α2δ2 subunit functions as a developmental switch that limits axon growth and regeneration while favorably controlling synapse formation. Mice with crush SCI showed enhanced regeneration of CST and ascending sensory axons when α2δ2 was pharmacologically blocked with gabapentinoid [138,139,140].

### 4.7. Neuromodulation and Brain–Spine Interfaces

When combined with physical rehabilitation, lumbar spinal cord stimulation (SCS) has improved stepping and ambulation in patients with chronic SCI. After a week of combined therapy, improvements in locomotion have been observed, with lasting improvements in ground walking at the 1-year follow-up [176,177].

In recent years, there has been an increase in the use of brain–computer interfaces (BCIs) to regulate upper extremity function, which includes reaching and gripping. For patients to benefit, it is crucial to bring this technology into a community setting that is closer to their homes. Either soft robotics (SR) or functional electrical stimulation (FES) constitute the technical foundation of the majority of these interactions [178].

Traditional FES therapy involves teaching patients to voluntarily contract their muscles to perform a predetermined task while surface-level or implanted electrodes from the FES system stimulate the muscles. According to this approach, conscious movements occur simultaneously with peripheral sensations and brain activity brought on by the conscious effort. The FES generates additional afferent input, which enhances practice-induced plasticity in the brain and spinal cord [179,180,181].

SR devices use soft actuators, which are frequently back-drivable, and flexible connections to improve comfort and flexibility while adapting to the natural curves of the human body. SR hand-function devices are lightweight and portable, making them easy to use at home for rehabilitation [182,183].

## 5. Conclusions

Early decompressive surgery, MAP optimization, and targeted rehabilitation have all been shown to be beneficial in the management of SCI. Current research in the area of pharmacological agents, cell-based therapies, endogenous regeneration and electrical stimulation aims to mitigate different components of the secondary injury cascade as well as to enhance damage repair mechanisms. The key to improvements in long-term patient outcomes will be the translation of these interventions and their subsequent application in the care of traumatic SCI patients.

## Figures and Tables

**Table 1:** Summary of the AO Spine and Praxis guidelines for the timing of surgery and hemodynamics for SCI.

**Timing of Surgery for SCI**			
**Key Question**	**Recommendation**	**Quality of Evidence**	**Strength of Recommendation**
Should we recommend early decompressive surgery (≤24 h after injury) for adult patients with acute SCI regardless of injury severity and neurological level?	We recommend that early surgery be offered as an option for adult patients with acute SCI regardless of level [22].	Moderate	Strong
Should we recommend ultra-early decompressive surgery for adult patients with acute SCI regardless of injury severity and neurological level?	A recommendation for ultra-early surgery could not be made on the basis of the current evidence because of the small sample sizes, variable definitions of what constituted ultra-early and the inconsistency of the evidence [22].	NA	NA
**Hemodynamics for SCI**			
**Key Question**	**Recommendation**	**Quality of Evidence**	**Strength of Recommendation**
Should we recommend the augmentation of MAP to at least 75–80 mmHg and not higher than 90–95 mmHg in order to optimize spinal cord perfusion in acute traumatic SCI?	We suggest the augmentation of MAP to at least 75–80 mmHg but not higher than 90–95 mmHg in order to optimize spinal cord perfusion in acute traumatic SCI [23].	Very Low	Weak
Should we recommend the augmentation of MAP for a duration of 3–7 days in order to optimize spinal cord perfusion in acute SCI?	We suggest the augmentation of MAP for a duration of 3–7 days in order to optimize spinal cord perfusion in acute SCI [23].	Very Low	Weak

**Table 2:** Summary of emerging pharmacological therapies for SCI.

Drug	Mechanism	Dosing	Comments
**Methylprednisolone (MPSS)**	Acts as a corticosteroid and antioxidant that reduces inflammation and improves spinal cord blood flow by blocking lipid peroxidation and lowering calcium influx.	30 mg/kg bolus followed by 5.4 mg/kg/h infusion × 24 h	The NASCIS II noted an increase in patients’ motor score by 4.8 points compared to placebo [69], while in the NASCIS III the group that received 48 h of MPSS showed an additional 6 points of motor improvement compared to the 24 h MPSS category for patients whose therapy was started 3 to 8 h after injury [70].
**Riluzole**	Blocks sodium channels to reduce excitotoxic injury and increases the preservation of glutamatergic synapses and motor neurons caudal to the lesion site, resulting in improvements in locomotor and respiratory function.	100 mg twice per day (BID) for the first 24 h, followed by 50 mgBID × 13 days	The Riluzole in Spinal Cord Injury Study (RISCIS) noted that cervical SCI patients (AIS grades A, B, and C) treated with riluzole demonstrated improvements in the functional recovery, which were part of the preplanned secondary analyses [71].
**Anti-Nogo-A Antibody**	Antibody against Nogo, thereby inhibiting the Rho-ROCK pathway and facilitating axonal regeneration following SCI.	5 to 30 mg/2.5 mL/day	Acute SCI patients who received the anti-Nogo-A antibody (ATI355) through intrathecal injection demonstrated improvements in motor scores and had satisfactory antibody tolerance [72]. Currently being tested in the RESET trial (NCT03989440) for acute cervical SCI [73], and Nogo Inhibition in Spinal Cord Injury (NISCI) clinical trial (NCT03935321) for chronic cervical SCI [74].
**Anti-RGMa Antibody (Elezanumab)**	Antibody for Repulsive Guidance Molecule A (RGMa) that inhibits axon development by binding to the Neogenin receptor, triggering the RhoA-Rho kinase pathway.	NA	Currently being tested in the ELASCI clinical trial, which is aiming to evaluate the safety and efficacy of Elezanumab in traumatic cervical SCI patients [75].

**Table 3:** Summary of emerging regenerative therapies for SCI.

Therapy	Mechanism	Notes
Neural Stem Cells (NSCs)	Tripotent stem cells that have been shown to myelinate denuded axons and encourage tissue sparing.	A recently conducted phase II trial showed a positive trend for UEMS and GRASSP scores in the interim analysis of patients who were transplanted with human fetal-derived NSCs. However, the magnitude of the improvement was below the clinical efficacy threshold and the trial was terminated early [123].
Mesenchymal Stem Cells (MSCs)	Improve tissue sparing and stimulate angiogenesis via neurotrophic signaling and immunomodulation. When injected directly into the spinal cord, can control macrophage activity and encourage tissue sparing.	Several active clinical trials evaluating the safety, efficacy, and dosage of MSC produced from adipocytes, bone marrow and umbilical cord. (NCT03505034 [124]; NCT02481440 [125]; NCT03521323 [126]; NCT03308565 [127])
Oligodendrocyte Progenitor Cells (OPCs)	Improve motor recovery by reducing cavitation volume, enhancing white matter sparing, and increasing oligodendrocyte survival.	In the SCiStar trial, 95% (21/22) of patients with AIS grade A and B secondary to subaxial cervical spine recovered at least one motor level on one side, and 32% (7/22) recovered two or more motor levels on one side [128].
Schwann Cells	Express growth-promoting proteins and act as a structural framework to direct developing axons and myelinate regenerating axons.	A phase I study of 6 patients with thoracic injury noted no adverse outcomes connected to nerve harvesting or the transplant surgery. ASIA Impairment Scale (AIS) grade A to grade B clinical improvements noted were within the typical range for patients with thoracic SCIs. Subclinical improvements in motor cortical connections were observed in neurophysiological investigations (motor evoked potentials and electromyography) [129]
Endogenous Stem Cells	Located in the central canal region of the spinal cord, these are ependymal derived neural stem/progenitor cells that become activated after SCI and primarily differentiate into astrocytes, with a smaller subset differentiating into oligodendrocytes. Several studies have demonstrated that these cells are important in promoting axon regeneration, providing beneficial trophic support and contributing to baseline functional recovery.	Several studies underway to further enhance the activation of endogenous ependymal derived neural stem/progenitor cells and direct their fate specification [130,131,132,133,134,135].
Biomaterials/Scaffolds	Promote the development, survival, and plasticity of cells by introducing exogenous stem cells into the injured area and by providing an environment and scaffold for the regeneration of endogenous circuits.	The INSPIRE trial included 19 patients in whom neuro-spinal scaffolds were implanted 96 h after injury. At 24 months, there were no long-term neurological complications, and at 12 months and beyond, favorable AIS conversions were observed [136].
Upregulating Intrinsic Regenerative Potential	Manipulating cytoskeletal dynamics, ion channels, and signaling pathways that ultimately impact reactivation of intrinsic growth programs and promoting axon regeneration.	Taxol and epothilone B promote microtubule polymerization and reduce fibrotic scarring, thereby inducing axon regeneration and improving function [137]. α2δ2 blockage achieved with gabapentinoid has been shown to enhance CST growth and improve motor function [138,139,140].

## Data Availability

Not applicable.
